# Mentoring for early-career women in health research: the HIGHER Women Consortium approach

**DOI:** 10.1017/gheg.2016.20

**Published:** 2017-03-06

**Authors:** S. K. Kwedi Nolna, P. E. Essama Mekongo, R. G. F. Leke

**Affiliations:** 1 Faculty of Medicine and Biomedical Sciences, University of Yaoundé I, Yaoundé, Cameroon; 2 Capacity for Leadership Excellence and Research, Yaoundé, Cameroon; 3 Faculty of Law and Political Science, University of Yaoundé II, Yaoundé, Cameroon; 4 The Biotechnology Center, University of Yaoundé I, Yaoundé, Cameroon

**Keywords:** Career development, higher education, mentoring, researchers, women

## Abstract

Attracting and retaining women in health research is crucial as it will maximize creativity and innovation as well as increase gender competency and expertise in the field. To help address the gender gap in the research for health field in Cameroon, some women research scientists formed the Higher Institute for Growth in HEalth Research for Women (HIGHER Women) consortium to support and encourage the growth of women research scientists through a training institute with a Mentor–Protégé Program (MPP). The consortium set up a MPP aiming at providing professional guidance to facilitate protégés' growth and emergence in health research. The consortium has conducted two workshops aiming at increasing the early-career women's skills needed to launch their career and focusing on proposal writing with the aim of producing a fundable project. Since 2015, the consortium has brought together approximately 100 women comprising of 80 protégés. The most significant outcome is in the protégés' feedback from their annual evaluations. The protégés are now more likely to submit abstracts and attend international conferences. Some grants have been obtained as a result of the working relationship with mentors. The HIGHER women consortium works to develop a pipeline of women leaders in health research by fostering growth and leadership culture through their MPP.

## Introduction

As a resource-limited sub-Saharan country, Cameroon tremendously needs health research. Communities are burdened with diseases, an inadequate health system and poverty. In order to combat these ordeals, the country must equip itself with manpower capable of achieving full potential and maximize talent. Stakes are too high for any portion of the talent pool to be underutilized. According to the Ministry of Women Empowerment and the Family, only 5.9% women have attained the full professor grade, the highest academic rank in higher education [1]. In general, the road to academic and research success is full of obstacles. Women researchers particularly have difficulties to attract funding for their research projects, access national and international collaborations and networks, reach leadership positions and publish as first and senior authors [2]. To help address this gender gap in Cameroon, a group of women research scientists formed the Higher Institute for Growth in HEalth Research for Women (HIGHER Women) consortium to support and encourage the growth of women research scientists through a training institute with a Mentor–Protégé Program (MPP). The expected impact is the advancement of women in health research in Cameroon thus triggering additional interest of younger women.

The HIGHER Women consortium opted for a Mentor–Protégé strategy utilizing female mentors willing to spare time to accompany early-career women through their professional journey. The protégé benefits from the guidance of a seasoned woman scientist who travelled a path similar to the one aspired and has accomplished a high level of professional success such as attaining leadership position in the field. The mentor–protégé relationship is also beneficial to the mentor who gets satisfaction in transferring knowledge and insights while being exposed to fresher perspectives. Mentoring also gives the experienced scientists the chance to exercise leadership and coaching skills. Mentors and protégés mutually benefit from being part of each other's network, which amplifies their visibility and expands their research networks.

Mentoring is an important approach that encourages women's professional development and has proven to be effective in other settings. Regardless of gender, mentors and role models have a positive influence on career advancement [3]. In March 2015, the HIGHER Women consortium obtained a small grant from the Special Programme for Research and Training in Tropical Diseases (TDR) for a project on women researchers career development. In July 2015, the International Development Research Centre (IDRC) Canada, approved additional funding to support the project which allowed the consortium to expand the workshop participation to 52 early-career women along with a follow-up workshop focusing on proposal development. The workshops aimed at: teaching early-career women researchers how to create a plan for attaining leadership goals; developing skills in seeking funds for innovative quality research projects; highlighting the challenges of building a career in health research and to devise methods for overcoming those challenges; and setting up networking opportunities with women role models that make a difference in health research in Cameroon.

Attracting and retaining more women in health research is crucial as it will maximize creativity and innovation as well as increase gender competency and expertise to conduct high-quality research. It is crucial that women learn to be strategic and trace a clear path for career development. By taking time to plan career growth, learn indispensable skills (such as fundraising for research projects), establish mentor–protégé relationships with role models, women are more likely to seize and hold on to leadership roles.

In this paper, we describe the HIGHER Women approach to mentoring, focusing on the early-career women's experiences, the project outcomes and how this program fits with and influence policy.

## Methods

### Setting up the MPP

The overall objective of the HIGHER Women's MPP is to provide professional guidance to facilitate protégés' growth and emergence in health research. Specifically, the HIGHER Women MPP aims at: advancing the professional development of women in health research to attain long-term sustainability in their careers; contributing to the development of quality practices in health research; increasing the women's ability to mobilize sizeable funding to execute quality research projects; increasing national and international visibility of Cameroonian women researchers through consistent, escalating and sustained research productivity; and enabling transition from protégé to mentor after successful completion of 5 years in the MPP.

A typical mentor is a well-established, successful woman health researcher willing to commit time, resources, and expertise to teach, develop and grow the career of emerging women in the field. The Mentor is usually a woman who has experience nationally or internationally currently working on a research project or has worked extensively in the field. The Mentor serves as an advisor to the protégé without compensation. Protégés are emerging women health researcher, who signed a MPP engagement form, working in health research in a Cameroonian research institution with 0–5 years experience.

The HIGHER Women consortium conducts annual evaluations of the MPP by reviewing annual reports submitted by mentor–protégé couples. This evaluation focuses on the progress and accomplishments realized.

### Consortium activities

#### The August 2015 Workshop

In April 2015, a call for applications was published in Cameroon's major media inviting applications from early-career women scientists in health research working in Cameroon. An evaluation committee comprising members of the consortium selected 52 women out of 86 applicants. The selection was based on relevance and quality of the Curriculum Vitae (CV), applicant's motivation, appropriateness of the applicant's career plan, professional experience and age.

The workshop brought together 50 participants (two selected applicants were excused), six workshop assistants, three male facilitators and 17 mentors representing 18 research institutions and universities across the country.

The event was supported by the three ministries in charge of research in Cameroon: the Ministry of Higher Education, the Ministry of Research and Innovation and the Ministry of Public Health. High government officials from these ministries attended the opening ceremony chaired by the Minister of Higher Education.

The workshop started with sessions on research fundamentals such as general research methodology, qualitative research methods, quantitative methods, research ethics, scientific integrity, intellectual property and protocol writing. On the 2nd and 3rd days, participants had sessions in soft skills such as project management, goal setting, career plan design, motivational letter writing, visibility of work, Career-enhancing use of social media, Leadership, Time management, ‘Tricks of the trade’, Publish or perish and Translating research into policy. The 4th day was dedicated to ‘shadowing a female research scientist’ giving participants the opportunity to spend an entire day following female research scientists in their laboratories and research institutions. On the last day, participants presented on their five days workshop experience: their motivation for participating in the workshop, their most memorable moment, the work done with their mentors and how they planned to apply acquired skills. The closing ceremony was chaired by the Minister of Women Empowerment and the Family, a woman who made her mark as renowned Pediatrician and Researcher.

#### The July 2016 Workshop

A second workshop was held on 6–8 July 2016 with 28 of the women who attended the August 2015 workshop joined by 12 new recruits to focus on how to write successful research proposals likely to attract financing. Early-career women were selected through a competitive process consisting of reviewing research project synopses, letters of intent and CV. The workshop accommodated 40 women out of more than 64 candidates who responded to the call. After the workshop, attendees were expected to have a fully written competitive research proposal. Attendees were also expected to identify relevant, current and open calls and submit their proposals.

The first day of the workshop was dedicated to a review of proposal writing fundamentals: review of a proposal's components, research methodology, generating budgets, timelines, formulating expected outcomes, writing biographical sketches/CVs and project evaluation.

The second day was dedicated to the actual proposal writing. Groups of five to six participants worked with one mentor. Each individual worked to transform a three pages synopsis into a full proposal. Protégés worked out proposals' blocks with their mentors and also benefited from bouncing ideas with their peers.

The workshop ended with each group presenting a recap of the first 2 days' accomplishments. Certificates were handed to participants at the closing ceremony.

### Fireside conversations

The HIGHER women consortium takes a holistic approach to mentoring that includes personal growth. The consortium recognises that barriers to women's road to professional success are biological, cultural, sociological and historical. During both workshops, mentors and protégés gathered around a fire in evenings for ‘Fireside Conversations’ reminiscent of an old African tradition. Sitting and talking around the fire created intimacy and trust, and promoted open exchange. Mentors freely shared the difficulties they faced and how they balanced personal lives and careers. Talks were inspirational and provided lessons-learned to early-career women.

## Outcomes

The most significant outcome from this project is in protégés' feedback as obtained from workshops' evaluations. The following quotes were written by the HIGHER Women protégés.

“Since last year, HIGHER Women consortium has greatly impacted my life. I am now in the habit of sending applications for opportunities that come my way. HIGHER gives me a network of researchers that I can count on. For example I contacted one of the protégés when I was in need of some microorganisms for my laboratory work, and she directed me on how to obtain them. The proposal synopsis I submitted for the workshop was actually in a domain that is still a challenge to me but I dared to conceive and design it in partnership with a master student that I am supervising. The achievements of others in our consortium also give me hope and now I know that I can also make it and I will make it.”

“HIGHER Women helped me to see the need to apply for a PhD program. It has also helped me to build confidence in myself as a health researcher. Thank You to all the Mentors.”

“I am a French speaker and HIGHER women has encouraged me to work on my English as they said that English is the language of science. I wrote my workshop application, cover letter and curriculum vitae in English although it was a big challenge for me.”

“I appreciate the elder/younger relationship with my Mentor. The motivation from the achievements of other HIGHER women, announcements sent in our email boxes, celebration of family events, the feeling of family we get from each other and specially the joy of togetherness.”

“I have more confidence in myself. I better manage my time and I organize my work. I submitted an abstract that was selected in two international conferences.”

“The motivation and moral support I get from the HIGHER Women is very envigorating and it makes me venture for activities I was not able to do before. I also appreciate the fact that I can approach another mentor if my mentor is unavailable. I am proud to belong to an association that strives to improve and encourage women to be their best. I shared this experience in a conference I attended in Japan and this was highly appreciated.”

“I have taken 100% responsibility of my research work. I have an acceptance letter for the TWAS fellowship. I am able to knock on any Professor's door at the faculty to ask for help thanks to networking skills I learnt. I am learning French language because HIGHER women presenters challenged me with bilingualism during presentations. I started an online course on public speaking because HIGHER Women taught the need to communicate my research findings with the world. I have become a model to many young female students in my faculty. I have become very conscious and encouraging to young girls in secondary school where I work as a chemistry teacher. Every aspect of my life has experienced positive impact when I listened to stories shared at the fireside conversations mentors, I understood that all women had challenges in climbing the academic ladder. Even my greatest role model in research passed through challenges to get to her grade of Professor Emeritus. The HIGHER Women consortium is a gold mine to me.”

“HIGHER Women has really helped me understand a lot in the field of research and the role of good research and its impact. It opened my eyes to opportunities as far as participating and forwarding abstracts for international conferences. It was the gateway for me as far as PhD fellowships and funding opportunities are concerned. I also learned about success for a woman in the context of the African society.”

## Recommendations

Moving forward, the following recommendations can be made:

- Motivate the mentors by providing them with resources that will help them in organizing face to face meetings with their protégés such as network socials and small group workshops.
- Work with the universities and research institutions to promote the recruitment, retention, advancement and leadership of women in health research throughout their careers.
- Organize a central African forum (include other networks in Gabon, Congo, Equatorial Guinea, Chad and Central African Republic) to network, collaborate, develop policy, engage in research and provide faculty development sessions pertaining to advancing and advocating for women in health research.

## Conclusion

The field of health research will benefit from the development of a cadre of knowledgeable, experienced and energized women having learned to be role models for future generations. This is in line with government policy to reduce unequal access of girls/women to education broadly and STEM specifically as contained in the National Gender Policy Document 2011–2020 in Strategic areas 1 and 5, which focuses on enhancing women's participation and representation in public life and decision-making. The HIGHER women consortium keeps working to develop a pipeline of women leaders in research by fostering growth and leadership culture through their MPP. With a sharp plan, support, motivation and inspiration, the future of health research in Cameroon is for women to grab and shine.
